# Investigation on the hydrolytic mechanism of cucurbit[6]uril in alkaline solution

**DOI:** 10.1098/rsos.180038

**Published:** 2018-05-02

**Authors:** Chao Zhu, Zihui Meng, Wenjin Liu, Hongwei Ma, Jiarong Li, Tongtong Yang, Yang Liu, Ni Liu, Zhibin Xu

**Affiliations:** 1 School of Chemistry and Chemical Engineering, Beijing Institute of Technology, Beijing 100081, People's Republic of China; 2 School of Chemical Engineering, Yunnan Open University, Kunming 650223, People's Republic of China

**Keywords:** cucurbit[6]uril, hydrolysis reaction, product separation, mechanism research

## Abstract

The structure of cucurbit[6]uril (CB[6]), as a fascinating supramolecular receptor, is regarded as ‘indestructible’. Herein, we investigated the hydrolysis of CB[6] catalysed by alkali. Our results showed that CB[6] was easily hydrolysed in 30% NaOH at 160°C within 3 h. Separation and purification of hydrolytic products demonstrated the presence of NH_3_, CO_2_, HCOONa, glycine and hydantoic acid. Based on the studies of the hydrolysis of substances similar to CB[6] including 4,5-dihydroxyethyleneurea, glycoluril and glycoluril dimer, we proposed that a plausible reaction mechanism involved a Cannizzaro reaction, which is supported by HPLC, mass spectrometry data and previous reports. Further studies are dedicated towards a controlled hydrolysis of CB[6], which will provide a new route for direct functionalization of CB[6].

## 1. Introduction

Cucurbit[*n*]urils (CB[*n*]) were first synthesized in 1905 and characterized by X-ray diffraction in 1981 [1,2]. Subsequently, the CB[*n*] family rapidly expanded with the discovery of new members including CB[10] [3,4], CB[14] [5], CB[13] and CB[15] [6]. Because of their robust structure and hydrophobic cavity, CB*[n*] compounds hold great promise in supramolecular chemistry. For instance, they have been applied in molecular machines [7], sensing ensembles [8,9], drug delivery [10–12] and biomimetic systems [13,14]. However, poor solubility of CB[*n*] compounds in water and common organic solvents has limited their practical applications [15–17]. Thus, numerous analogues and derivatives of CB[*n*] have been designed and synthesized [18–22]. Nevertheless, it has proved challenging to achieve direct functionalization of CB[*n*] compounds due to their high stability. In 2003, Kim and co-workers [22] first reported the reaction of CB[6] with K_2_S_2_O_8_ producing perhydroxycucurbit[6]uril. Based on Kim's research, Li and co-workers further investigated the type of oxidant and optimized the reaction conditions, but it was still very difficult to control the depth of oxidation [23]. The synthesis of monofunctionalized CB[*n*] in a controlled environment also has several successful examples. With the help of guest molecules and theoretical calculation, Scherman and co-workers [24,25] and Bardelang and co-workers [26] were able to produce monohydroxylated CB[*n*], which possessed better solubility and modificability, and thus have been successfully used in protein extraction [27], adhesives [28] and drug transportation [29]. Although there are many reports about direct oxidation of CB[*n*], hydrolysis of CB[*n*] for further modification has not been reported directly.

**Scheme 1.**
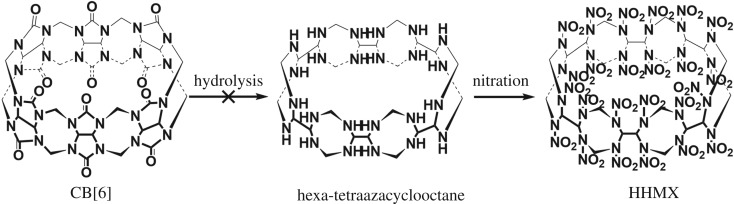
Designed route for the preparation of HHMX from CB[6].

Hexamer octogen (HHMX), a derivative of CB[6], possesses a macrocyclic crown structure. It also exhibits, in theory, a detonation velocity of 10 500 m s^−1^, a detonation pressure of 50 GPa and a density of 2.11 g cm^−3^, representing a better choice than one of most powerful explosives hexanitrohexaazaisowurtzitane (CL-20) [30,31]. As an excellent precursor for the preparation of HHMX, CB[6] was designed to produce HHMX by hydrolysis and nitration (scheme 1). In this work, we report investigations for the hydrolysis of CB[6] using a number of catalysts. Our research showed that strong base was able to hydrolyse CB[6] to give NH_3_, CO_2_, HCOONa, glycine and hydantoic acid, and accordingly, a feasible reaction mechanism was proposed and verified. Although no expected products were obtained, the results still provide a great amount of valuable information to aid further research on the controlled hydrolysis of CB[6].

## 2. Material and methods

### 2.1. Chemicals and instruments

All materials were used as analytical pure grade or higher and purchased from local suppliers without further purification. Urease was purchased from Sigma (SP, 100 KU g^−1^). NMR spectra were recorded on a Bruker Avance II 600 MHz spectrometer with TMS as an internal standard. High-resolution mass spectroscopy (HRMS) was performed on an Agilent Q-TOF-MS 6520. The X-ray crystal structure determinations were performed on a Bruker D8 Venture. HPLC analyses were performed using a Shimadzu LC-20 system equipped with an auto-sampler and a diode array detector. Glycoluril, 4,5-dihydroxyethyleneurea, glycoluril dimer and CB[6] were readily prepared according to our previous work [32], Kim *et al*. [18] and Svec *et al*. [33].

### 2.2. General procedure for the hydrolysis reaction and separation

Cucurbit[6]uril, glycoluril, 4,5-dihydroxyethyleneurea and glycoluril dimer (2.0 g) were added to sodium hydroxide solution (6 g of NaOH in 14 ml of H_2_O). The mixture was then heated in a sealed hydrothermal synthesis reactor at 160°C for 3 h. After the reactor was cooled to room temperature, the reaction mixture was acidified with 37% HCl aqueous solution to pH 7. The mixture was then concentrated under reduced pressure until no more solvent could be distilled off; to afford a yellow solid. The resulting solid was dissolved in methanol and concentrated in a vacuum, this was repeated three times (30 ml each time) to remove NaCl and NH_4_Cl. The residue was purified by column chromatography with CH_3_OH/CH_2_Cl_2_ (1 : 1.5) as the eluent to give HCOONa, glycine and ammonium hydantoate as end products. Structural characterization of the above-mentioned three products was confirmed by X-ray structure, NMR spectra and HRMS spectra (see electronic supplementary material).

## 3. Results and discussion

### 3.1. Hydrolysis of CB[6]

Generally, hydrolysis is carried out under enzyme catalysis, acid catalysis or base catalysis. As the hydrolysis of CB[6] has not been reported yet, we tried the above methods systematically. The results are shown in table 1.

**Table 1.** Results of hydrolysis reaction of CB[6] in the presence of different catalysts. *C*, concentration (mole or mass fraction); *T*, temperature; *t*, time.

entry	class	catalyst	condition^a^	products
1	enzyme catalysis	urease	*T*: 37°C; *t*: > 7 days; pH 7.4	none
2	acid catalysis	HCl	*C*: 2 M – M_max_;	none
3		H_2_SO_4_	*T*: 100 – 180°C;	
4		HNO_3_	*t*: 6 – 12 h	
5	base catalysis	NH_3_·H_2_O	*C*: 27%; *T*: 10 – 180°C; *t*: 6 – 12 h	none
6		NaOH	*C*: 30%; *T*: 180°C; *t*: 6 h	irritant gas, suspension became clear orange solution

^a^H_2_O as a solvent was used in each reaction and sealing tubes were used when *T* ≥ 100°C.

Urease can specifically hydrolyse urea and its derivatives such as hydroxyurea, releasing CO_2_ and NH_3_ [34]. Herein, urease was used as catalyst to hydrolyse CB[6]. The reaction was carried out at 37°C for more than 7 days at the optimum pH, but no product was obtained and hardly any weight loss of CB[6] was detected at the end.

Acid is a common hydrolysis catalyst, and thus the effects of different acids concentrations at different temperatures and reaction times were studied. Not surprisingly, there was no indication of a hydrolysis reaction occurring, because CB[6] was prepared from concentrated HCl and concentrated H_2_SO_4_ at high temperature. Hence, this is strong proof that CB[6] is extremely stable in acidic solution, even at high temperature (180°C), high pressure and strongly acidic conditions.

Base hydrolysis also was investigated. Only reaction using NaOH as catalyst resulted in transformation of the raw material. Thus, the effects of different factors on this reaction were investigated (table 2).

**Table 2.** Hydrolysis of CB[6] at different conditions in NaOH solution.

entry	C(NaOH) (% in mass)	*T*^a^ (°C)	time (h)	products^b^
1	10	180	12	none
2	20	180	12	small amount of irritant gas
3	30	80	12	none
4	30	100	12	small amount of irritant gas
5	30	120	12	irritant gas, colour of the reaction solution is deepened
6	30	140	5	lots of irritant gas, the reaction became dark brown
7	30	160	3	
8	30	180	2	

^a^H_2_O as a solvent was used in each reaction and sealing tubes were used when *T* ≥ 100°C.

^b^Reaction was monitored by thin layer chromatography to determine whether the reaction was carried out or completed.

As shown in table 2, no hydrolysis occurred when the concentration of NaOH was lower than 20% and temperature was lower than 100°C. When the temperature was increased to 140°C and the concentration was increased to 30%, the reaction time was significantly shortened and the conversion rate was significantly increased. Finally, the reaction conditions were established as follows: 30% wt NaOH aqueous solution at 160°C for 3 h.

### 3.2. Separation and characterization of hydrolytic products

**Scheme 2.**
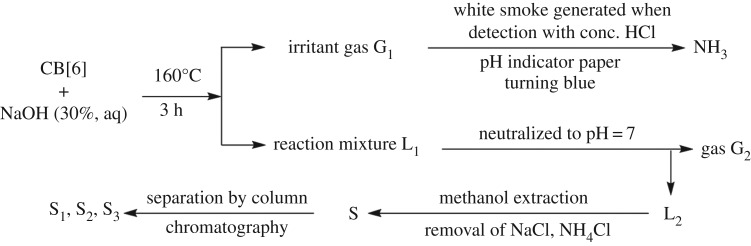
Separation flow chart of CB[6] hydrolysate.

We have studied the separation of hydrolytic products (scheme 2). Irritant gas **G_1_** caused the pH test paper to turn blue and made the glass rod soaked with concentrated HCl generate white smoke which was identified as NH_3_. There were white precipitates when gas **G_2_** was bubbled into the Ba(OH)_2_ solution. Simultaneously, the phenomenon of litmus solution turning red upon the inlet of **G_2_** definitely indicated that **G_2_** was CO_2_. The crude product **S** was obtained by repeatedly concentrating and dissolving **L_2_** to remove NaCl and NH_4_Cl using methanol as solvent. Ultimately, three solid products **S_1_**, **S_2_** and **S_3_** were obtained after purification by column chromatography.

**Figure 1.**
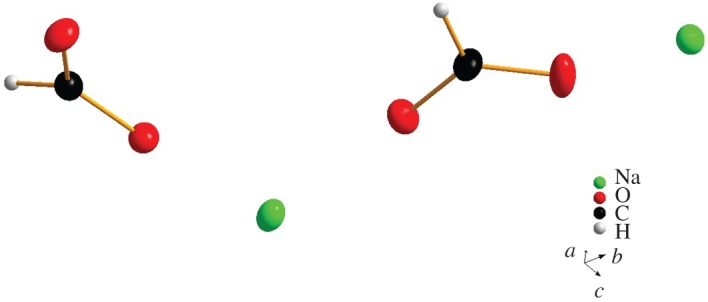
Molecular structure of HCOONa shown with 30% probability thermal ellipsoids.

NMR and mass spectrometry were applied to characterize the above products. As the results shown by NMR and mass spectrometry were relatively few and uncertain, their basic structures cannot be confirmed. However, single crystals suitable for X-ray crystal structure determination of compound **S_1_** were obtained by slow evaporation in H_2_O–CH_3_OH solution. The X-ray structure revealed **S_1_** to be HCOONa (figure 1), which was in agreement with NMR and MS analysis.

**Figure 2.**
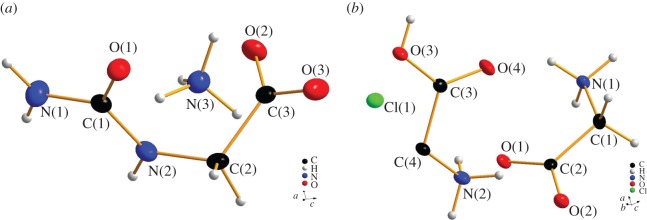
Molecular structure of ammonium hydantoate (*a*) and glycine (*b*) shown with 30% probability thermal ellipsoids.

**Scheme 3.**
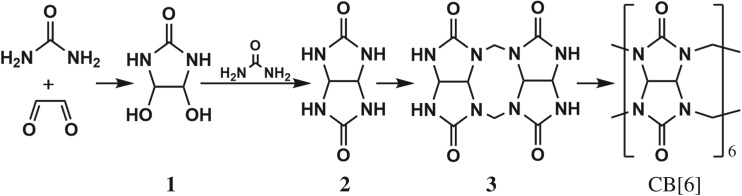
Synthetic route of CB[6].

So far, the three known products are NH_3_ (was certainly from the only nitrogen atom), CO_2_ (was likely to come from the carbonyl) and HCOONa (may be from methylene). To unravel the identity of products **S_2_** and **S_3_**, we studied the hydrolysis of three compounds, 4,5-dihydroxyethyleneurea (**1**), glycoluril (**2**) and glycoluril dimer (**3**) (scheme 3) that serve as precursors for producing CB[6], because these compounds have the basic structural unit of CB[6]. The hydrolysis of glycoluril (**2**) was readily completed in 20 min, and the hydrolysates were separated and purified via a separation method similar to scheme 2. In addition to NH_3_ and CO_2_, two main products **P_1_** and **P_2_** were also obtained. Single crystals of **P_1_** and **P_2_** were obtained in solutions of CH_3_OH by slow evaporation. X-ray crystal structures showed that **P_1_** was ammonium hydantoate and **P_2_** was glycine (figure 2). By comparing and analysing the NMR and HRMS data (table 3), it was not difficult to conclude that **S_2_** was glycine and **S_3_** was hydantoic acid. At the same time, we also found glycine and hydantoic acid in the hydrolysis of **1** and **3**, and that **3** produced HCOONa.

**Table 3.** NMR (D_2_O) and HRMS data of compounds **S_2_**, **S_3_**, **P_1_** and **P_2_**.

compounds	^1^H NMR (ppm)	^13^C NMR (ppm)	HRMS (*m/z*), *I* (%)
**S_2_**	3.53 (s, 1H)	175.06, 44.09.	76.0402 [M+H]^+^(6), 330.3374(100)
**S_3_**	3.70 (s, 1H)	188.35, 175.46, 44.52	117.0302 [M−H]^−^(69), 193.0319(100)
**P_1_**	3.79 (s, 1H)	183.91, 174.34, 43.82	74.0258(16), 117.0308 [M−H]^−^(100)
**P_2_**	3.51 (s, 1H)	176.23, 44.61	64.0166(17), 76.0396 [M+H]^+^ (100)

We detected the formation of urea by HPLC combined with HRMS upon the addition of xanthydrol into the hydrolytic mixture (see electronic supplementary material). At room temperature and acidic conditions, xanthydrol could readily react with urea to form a urea derivative with strong UV absorption [35]. This confirms that hydrolysis of glycoluril produces urea, which is another reaction intermediate.

### 3.3. Research on the mechanism of hydrolysis

In the presence of acid, CB[6] can be synthesized via intermediates **1**, **2**, **3** step by step (scheme 3). Considering the fact that **1**, **2**, **3** and CB[6] have the same products under the same hydrolysis reaction conditions, they must have experienced a similar reaction process. Therefore, CB[6] may also gradually hydrolyse to **1**, **2** and **3** under alkaline conditions. CB[6] has a great steric hindrance and the inert tertiary amine. Hence, it is the most difficult material to hydrolyse among the above-mentioned three compounds. HCOONa was only produced in the hydrolysis of **3** and CB[6], which indicated that it was derived from bridged methylene groups. Generally, C–C bonds are difficult to break, so the C–C bonds in glycine and hydantoic acid may only derive from the waist C–C bonds in the glycoluril unit.

**Scheme 4.**
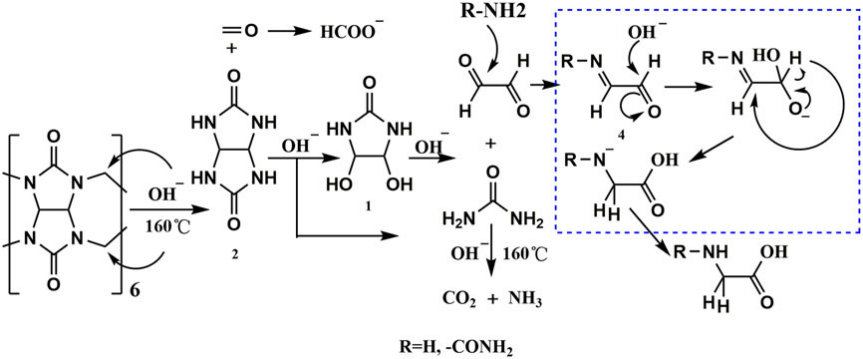
Designed hydrolysis mechanism for CB[6].

These observations led us to postulate a mechanism for the hydrolysis of CB[6] by NaOH (scheme 4). Under high temperature conditions, alkali first attacks the bridged methylene group, yielding formaldehyde and **2**. Formaldehyde can be oxidized by means of Cannizzaro reaction to produce HCOONa in the presence of concentrated NaOH. Subsequently, **2** undergoes ring-opening to form **1** and urea. Urea is easily decomposed into NH_3_ and CO_2_ at high temperature. Upon degradation of **1** into glyoxal and urea, NH_3_ and urea can condense with the glyoxal to give imide (**4**), thus resulting in glycine and hydantoic acid (Cannizzaro reaction), respectively.

**Figure 3.**
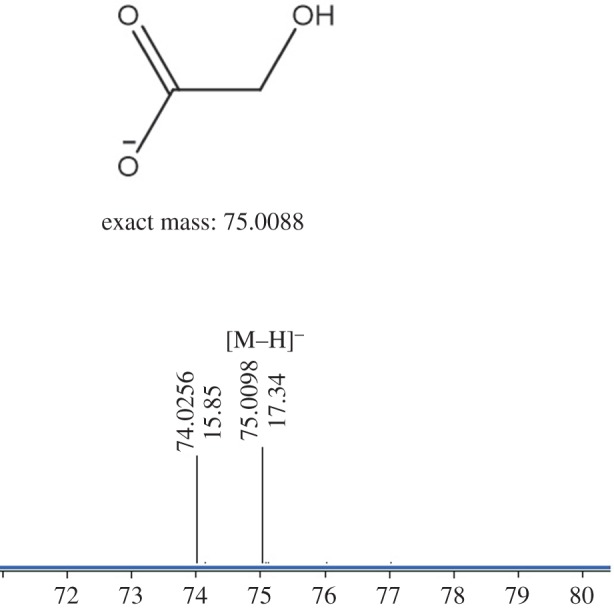
HRMS spectra of unseperated hydrolysate of glycoluril.

Oxidation of CB[6] could produce oxalic acid [23], and we found urea during the hydrolysis of glycoluril. These were both solid proof that these compounds were able to be hydrolysed to give glyoxal. As both glyoxal and imide (**4**) are extremely unstable under the reaction conditions, it is difficult to trap these intermediates. In addition to our main products, they would be quickly further converted to more stable compounds, such as hydroxyacetic acid, which can be synthesized from the intramolecular Cannizzaro reaction of glyoxal. Not surprisingly, under more moderate conditions, [M−H]^−^ ion peaks of hydroxyacetic acid were observed based on HRMS analysis during the hydrolysis of glycoluril (figure 3), which was indirect evidence of the appearance of glyoxal.

## 4. Conclusion

The catalyst urease cannot hydrolyse CB[6]. In reality, under high temperature (180°C) and high pressure, CB[6] can be stabilized in strong acid and weak alkaline solution, and only in strong alkaline solution will it slowly hydrolyse. Hydrolysis of CB[6] in 30% NaOH at 160°C for 3 h produces CO_2_, NH_3_, HCOONa, glycine and hydantoic acid. Combined with the results on hydrolysis of glycoluril, 4,5-dihydroxyethyleneurea and glycoluril dimer, a reasonable and feasible hydrolysis mechanism was proposed and verified by the related literature and HRMS. However, strong alkali hydrolysis destroys the skeleton structure of the whole CB[6], and research on controlled hydrolysis of CB[6] to achieve direct modification and obtain the target precursor for producing HHMX is still in progress.

## Data accessibility

The datasets supporting this article have been uploaded as part of the electronic supplementary material.

## Authors' contributions

C.Z., Z.M., J.L. and Z.X. conceived the ideas and designed methodology. H.M. and W.L. collected the data. T.Y. carried out the analyses. C.Z., Y.L. and N.L. contributed to the writing of the manuscript. All authors contributed critically to the drafts and gave final approval for publication.

## Competing interests

We have no competing interests.

## Funding

This work was funded by the Department of Science and Technology of Yunnan Province (Funding 2014FD041).

## Supplementary Material

Spectra from Investigation on the hydrolytic mechanism of cucurbit[6]uril in alkaline solution

## Supplementary Material

Crystallographic data in CIF from Investigation on the hydrolytic mechanism of cucurbit[6]uril in alkaline solution
