# Supplementary material for: Investigation on the hydrolytic mechanism of cucurbit[6]uril in alkaline solution
Source: R Soc Open Sci. 2018 May 2;5(5):180038. doi: 10.1098/rsos.180038 (PMC5990731; doi:10.1098/rsos.180038)
Supplement: Spectra from Investigation on the hydrolytic mechanism of cucurbit[6]uril in alkaline solution [file rsos180038supp1.docx]

**Supporting Information for**

**Investigation on the hydrolytic mechanism of cucurbit[6]uril in alkaline solution**

**Chao Zhu,^a,b^ Zihui Meng,^a^ Wenjin Liu,^a^ Hongwei Ma,^a^ Jiarong Li,^a^ Tongtong Yang,^a^ Yang Liu,^a^ Ni Liu,^a^ Zhibin Xu^*a^**

*a. School of Chemistry and Chemical Engineering, Beijing Institute of Technology, Beijing, 100081, The People's Republic of China*

*b. School of Chemical Engineering, Yunnan Open University, Kunming 650223, the People's Republic of China*

**CONTENTS:**

(A) The X-ray structure

(B) Copies of the NMR spectra

(C) Copies of the HRMS spectra

(D) Copies of the HPLC spectra

(E) Reference

**(A) The X-ray structure**

Crystal data for sodium formate (C H_1.25_ Na_0.75_ O_2_): *M* = 62.51, Triclinic, space group P-1, 0.200 x 0.200 x 0.200 mm^3^, *a* = 3.5301(6) Å, *b* = 8.4441(18) Å, *c* = 8.5228(18) Å, *α* = 66.421(7)°, *β* = 86.971(6)°, *γ* = 79.477(6) °, *V* = 228.87(8) Å^3^, *Z* = 4, *D_c_* = 1.814 g/cm^3^, *F*_000_ = 126, *λ* = 0.71073 Å, *T* = 296(2) K, 3538 reflections collected, 1135 unique [R(int) = 0.0694], Final GooF = 1.056, R1 = 0.0512, wR2 = 0.1347, R indices with I > 2sigma, 70 parameters, 0 restraints. Lp and absorption corrections applied, m = 0.480 mm^−1^, R indices (all data), R1 = 0.0710, wR2 = 0.1451.


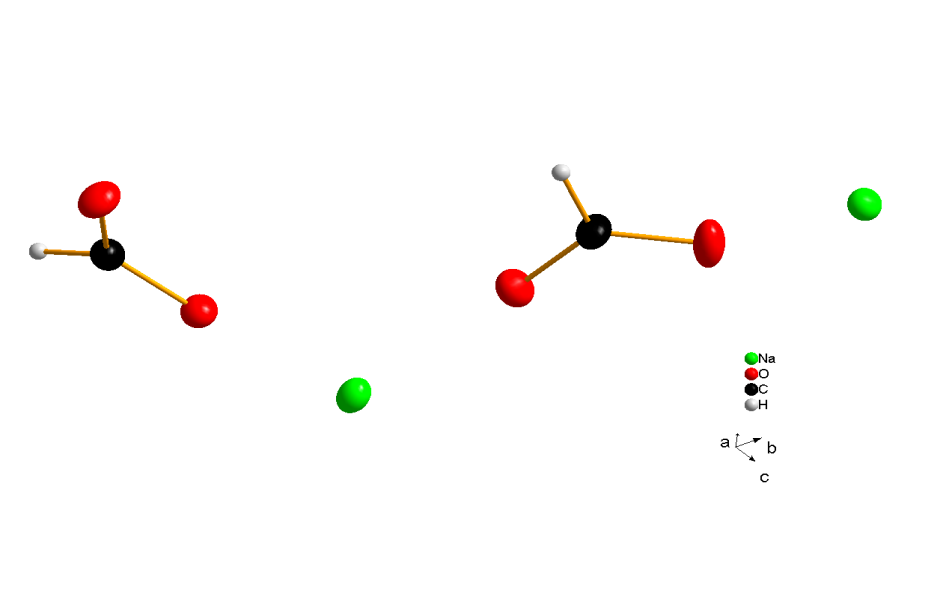


Crystal data for glycine (C_4_ H_11_ Cl N_2_ O_4_): *M* =186.60, monoclinic, Orthorhombic P2(1)2(1)2(1), 0.250 x 0.250 x 0.250 mm^3^, *a* = 5.3139(3) Å, *b* = 8.0975(4) Å, *c* = 18.0331(11) Å, *β* = 90°, *V* = 775.95(8) Å^3^, *Z* = 4, *D_c_* = 1.597 g/cm^3^, *F*_000_ = 392, *λ* = 0.71073 Å, *T* = 296(2) K, 15030 reflections collected, 1916 unique [R(int) = 0.0578], Final GooF = 1.020, R1 = 0.0367, wR2 = 0.0955, R indices with I > 2sigma, 101 parameters, 0 restraints. Lp and absorption corrections applied, m = 0.332 mm^−1^, R indices (all data), R1 = 0.0392, wR2 = 0.0970.


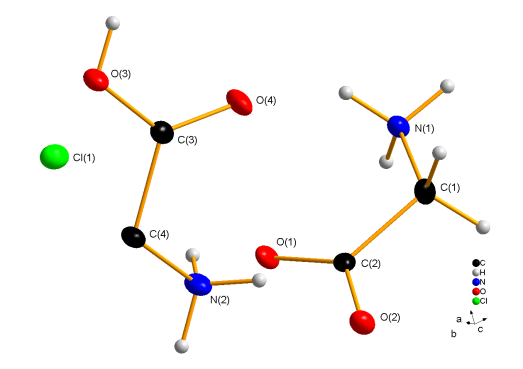


Crystal data for ammonium hydantoate (C3H9N3O3): *M* =135.13, monoclinic, space group P2(1)/n, 0.030 x 0.030 x 0.030 mm^3^, *a* = 4.8796(4) Å, *b* = 12.9910(9) Å, *c* = 9.8141(7) Å, *β* = 97.178(2)°, *V* = 617.25(8) Å^3^, *Z* = 4, *D_c_* = 1.454 g/cm^3^, *F*_000_ = 288, *λ* = 0.71073 Å, *T* = 296(2) K, 6762 reflections collected, 1514 unique [R(int) = 0.0517], Final GooF = 1.039, R1 = 0.0494, wR2 = 0.2671, R indices with I > 2sigma, 98 parameters, 0 restraints. Lp and absorption corrections applied, m = 0.291 mm^−1^, R indices (all data), R1 = 0.0569, wR2 = 0.1432.


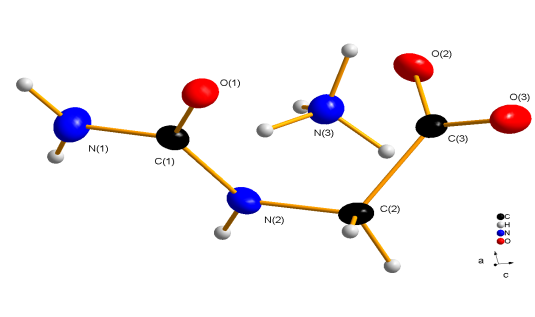


**(B) Copies of the NMR spectra**

**HCOONa**

**Glycine**

**Hydantoic acid**

**(C) Copies of the HRMS spectra**

**1. HRMS spectra of hydrolysis products**

a) HCOONa ([HCOO] ^-^): calcd. m/z 44.9977, found m/z 44.9998

b) Glycine ([M+H]^+^): calcd. m/z 76.0399, found m/z 76.0396

c) Hydantoic acid ( [M-H]^-^ ): calcd. m/z 117.0300, found m/z 117.0302

**b)**

**c)**

**a)**

**2. HRMS spectra of** **hydrolysis intermediates**

Urea ([M+H]^+^): calcd. m/z 61.0402, found m/z 61.0405

Hydroxyacetic acid ([M-H]^-^): calcd. m/z 75.0088, found m/z 75.0098

**(D) Copies of the HPLC spectra**

1. Samples.

Sample of glycoluril hydrolysis product was conducted as follows: glycoluril (2.0 g) and NaOH solution (10 ml, 30%) were reacted at 120 ℃ for 1 h, and neutralized with HCl (to pH = 7) after the reaction mixture cooled to room temperature, and then concentrated, dissolve with 20 ml CH_3_OH, and subsequently filtrated to give the sample solution.

The derivatization reaction was carried on as follows^1^: 0.4 mL of the xanthydrol solution (0.02 mol/L in 1-propanol) was added to 0.6 mL of a sample or standard, and then 0.1 mL of HCl (1.5 mol/L) was dropwise added, and finally reacted at room temperature for 30 min.

**2. HPLC method**

Chromatographic column: Venusil ASB C18 (4.6 mm × 150 mm, 5 μm); ACN/water: 40/60; flow rate: 1.0 mL/min; λ = 239 nm; Aliquots (10 μL) of sample solutions were injected; column temperature: 25 ℃.

**3. HPLC analysis of urea and its derivative**

(a). HPLC analysis of standard xanthydrol

(b). HPLC analysis of standard urea

(c). HPLC analysis of reaction mixture of standard urea and xanthydrol

(d). HPLC analysis of unpurified hydrolysis product

(e). HPLC analysis of reaction mixture of unpurified hydrolysis product and standard xanthydrol

**
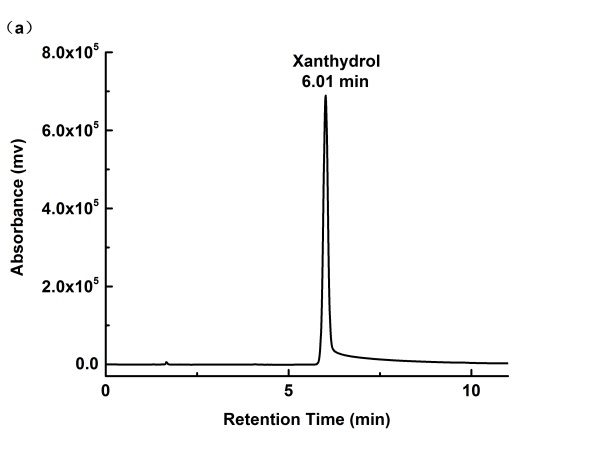

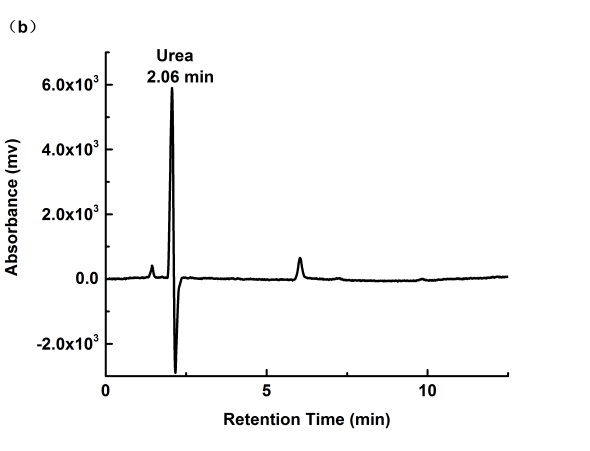

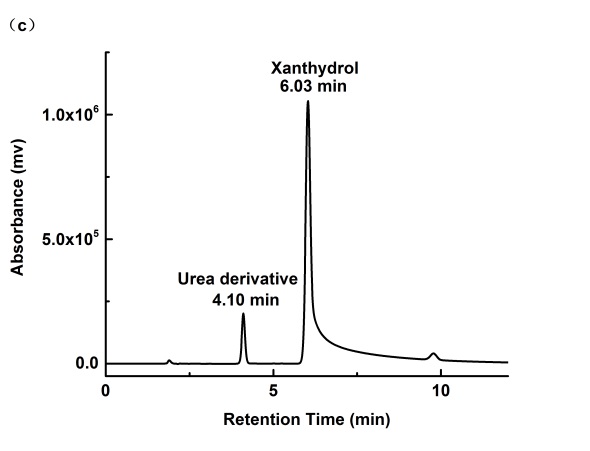

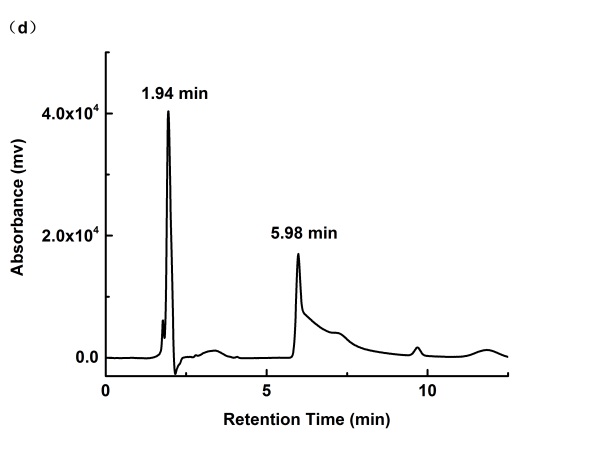

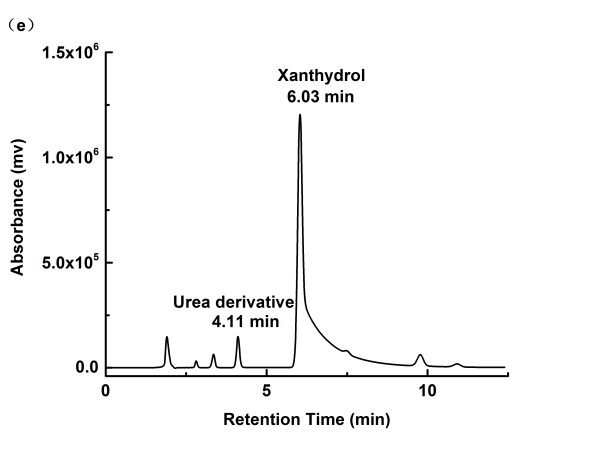
**

According to above chromatograms, the retention time for standard material were: 6.01 min (xanthydrol), 4.10 min (urea derivative). Urea (Fig. b) and hydrolyzed samples (Fig. d) almost didn`t have ultraviolet absorption at 239 nm. Comparing Fig. d with Fig. e, it can be found that there was an obvious peak of urea derivative (4.11 min) after hydrolyzed sample was treated with xanthydrol. Therefore, we could safely come to the conclusion that there was a formation of urea during the hydrolysis process.

**(E) Reference**

1. J. Zhang, G. Liu, Y. Zhang, Q. Gao, D. Wang and H. Liu, *Journal of agricultural and food chemistry*, 2014, **62**, 2797-2802.
